# A Comparative Study of Essential Oil Constituents and Phenolic Compounds of Arabian Lilac (*Vitex Trifolia* var. Purpurea): An Evidence of Season Effects

**DOI:** 10.3390/foods8020052

**Published:** 2019-02-02

**Authors:** Anahita Boveiri Dehsheikh, Mohammad Mahmoodi Sourestani, Paria Boveiri Dehsheikh, Sara Vitalini, Marcello Iriti, Javad Mottaghipisheh

**Affiliations:** 1 Department of Horticultural Science, Faculty of Agriculture, Shahid Chamran University of Ahvaz, Ahvaz 6135743311, Iran; anahitaboveiri84@gmail.com (A.B.D.); pboveiri17@gmail.com (P.B.D.); 2 Department of Agricultural and Environmental Sciences, Milan State University, via G. Celoria 2, 20133 Milan, Italy; Sara.vitalini@unimi.it; 3 Department of Pharmacognosy, Faculty of Pharmacy, University of Szeged, 6720 Szeged, Hungary; Imanmottaghipisheh@pharmacognosy.hu

**Keywords:** Verbenaceae, isoprenoids, β-caryophyllene, flavonoids, anthocyanins, antiradical capacity, DPPH

## Abstract

To evaluate the fluctuation of secondary metabolites in Arabian lilac during a year, aerial parts of the plant were harvested in the middle of each month. The essential oils content from fresh and dried plant materials was analyzed by gas chromatography-flame ionization detector (GC-FID) and gas chromatography-mass spectrometer (GC-MS), individually. Phytochemical contents, along with antiradical scavenging potential of the related methanol extracts were separately assessed. The spring and autumn samples (fresh and dried) yielded more essential oil than the other samples. Forty-one compounds were identified totally in the oils and the major constituents characterized were β-caryophyllene, sabinene, and caryophyllene oxide. The extracts obtained from winter and summer plants possessed the highest total phenolics. The maximum amount of total flavonoid content was measured in winter (December and January), whereas the minimum one was observed in spring (March). The summer and winter samples showed the highest and lowest content of flavones and flavanols, respectively, whereas the anthocyanin content was higher in winter than in summer. Moreover, antiradical activity of the extracts in summer and winter samples was higher than in other seasons. Overall, this study can provide useful information regarding the best harvest period of Arabian lilac to yield the desired compounds for application in phytopharmaceutical and food industries.

## 1. Introduction

The *Vitex* genus (Verbenaceae family) comprises nearly 270 species predominantly of trees and shrubs that are widely distributed in tropical and subtropical regions, along with some species growing in the temperate zones [1]. Many species of this genus have been extensively commended because of notable therapeutic effects on several female disorders such as endometriosis, abnormal menstrual cycle, menopausal conditions, corpus luteum insufficiency, hyper-prolactinaemia, infertility, acne, menopause, disrupted lactation, and cyclic breast pain, and, therefore, *Vitex* genus has been known as female herb since ancient times [2]. Furthermore, this genus possesses anti-inflammatory, anti-histaminic, anti-microbial, anti-pyretic, analgesic, and antioxidant activities and is used to remedy asthma, allergies, skin illnesses, diarrhea, and gastrointestinal and liver diseases. It was also reported these plants are insect repellent and aid in snake bite treatment [3]. The hydroalcoholic extract of *Vitex trifolia* exhibited the most potency against *Culex quinquefasciatus* larvae compared with other studied *Vitex* species [4].

The aforementioned benefits are attributed to the existence of a wide array of active substances including essential oils (EOs), phenolic acids, flavonoids, lignans, and anthraquinones, etc. [5].

*Vitex trifolia* L. is a stout aromatic shrub (less than 5 m height) with green-gray trifoliate leaves and white and violet flowers widespread in Southeast Asia, Micronesia, Australia, and East Africa [2,6]. *Vitex trifolia* var. purpurea commonly known as Arabian lilac is one of the most important varieties of this species. This plant can be easily distinguished from other plants of this species due to the annual color variation of leaves from green to violet and, therefore, it is mainly used as an ornamental plant in the landscapes. Leaves are traditionally recommended for the treatment of rheumatic pains, inflammation, sprains, and wound healing [1]. Infusion and decoction are also beneficial therapeutic preparations in the improvement of intestinal ailments and treatment of tuberculosis and amenorrhea and have been used by indigenous populations. Moreover, the essential oil (EO) of *Vitex trifolia* L. is often used as a sedative, an anti-inflammatory and used for headaches, colds and coughs as well as in liver disorders and HIV [1,7]. Phytochemical studies revealed that the methanolic extract of leaves possesses strong antioxidant activity and is considered as a potent anticancer agent due to its high content of phenolic compounds including phenolic acids, flavonoids, flavones, and flavonols [5].

In plants, the biosynthesis of secondary metabolites is strongly influenced by endogenous (genetic) and exogenous (environmental and edaphic) factors; therefore, their amount and chemical diversity are not constant during the plant lifecycle [8]. Environmental conditions, such as seasonal variations, are key factors capable of affecting the quantity and quality of these compounds. 

There is a high correlation between the content and chemical composition of EOs and phenolic compounds and the antioxidant capacity of some medicinal plants with seasonal variations. For instance, the evaluation of the quantity and quality of EOs extracted from different species of *Ocimum* and *Thymus* genus, *Origanum syriacum*, *Mentha pulegium*, *Thymbra spicata*, *Satureja thymbra*, *Salvia trilobal*, and *Lanata camara* has shown a wide range of variations during the different months and seasons [9,10,11,12,13,14,15]. The same correlation was reported for total phenolic and flavonoid contents as well as the antioxidant capacity of the *Thymus* genus and *Vaccinium myrtillus* with the seasonal variations in environmental conditions [9,11,16]. However, to the best of our knowledge, to date, no information has been reported on the effect of seasonal variations on the quality and quality of secondary metabolites of *Vitex* genus. Hence, the present study focused, for the first time, on the variability of the chemical profile of Arabian lilac in different months of a year, to determine the best period of harvesting and achieve the highest level of desirable bioactive compounds for uses in pharmaceutical and food industries.

## 2. Materials and Methods

### 2.1. Plant Materials

Aerial parts of Arabian lilac (*Vitex trifolia* var. purpurea) were collected in the middle of each month during the year 2014 from Ahvaz, a city in the south of Khuzestan province, Iran (latitude 31° 20’ N, longitude 48° 40’ E, altitude 20 m asl) located in an arid to semi-arid region with temperate winter and very hot summer (Table 1). Plant material was taxonomically identified by the botanist Dr. Mehrangiz Chehrazi and voucher specimens were deposited in the herbarium (KHAU-235). One kilogram of plant material was collected from five trees close to each other during the year. Samples were harvested from all (four) sides of each plant. The samples were mixed; half (500 g) was used to extract EO and stored in liquid nitrogen to determine total anthocyanin content. The second portion (500 g) was shade dried at room temperature (20–25 °C) to extract the EO of dried aerial parts and measure total phenolic, flavonoid, flavone, and flavanol contents and antiradical capacity.

### 2.2. Essential Oil Extraction

Fresh aerial parts (50 g) were individually subjected to hydro-distillation using Clevenger-type apparatus for 3 h according to the method recommended in the European Pharmacopoeia [17]. The obtained EOs were dried over anhydrous sodium sulfate (Na_2_SO_4_) and kept in sealed glass vials at 4 °C. The yields of EOs were determined based on fresh and dried matter and calculated as weight of oil (g) /100 g of fresh and dried aerial parts (% *w/w*), respectively. 

### 2.3. Essential Oil Composition

The essential oils were analyzed by Agilent 7890 gas chromatograph coupled with an Agilent 5975 mass spectrometer; HP-5MS (5%-phenyl–95%-methyl polysiloxane) capillary column (30 m × 0.25 mm i.d., 0.25 µm film thickness, Agilent technologies, Santa Clara, CA, USA); helium carrier gas at 1.5 mL/min; injector temperature 280 °C; detector temperature 300 °C; column temperature 40 °C (1 min)–300 °C (3 min) at 5 °C/min. The injection was done with a split ratio of 10:1. Scanning (1 scan/s) was accomplished in the range 50 to 500 *m/z* using electron impact ionization at 70 eV. The gas chromatography-flame ionization detector (GC-FID) analyses were performed with a Varian 3800 gas chromatograph, equipped with a flame ionization detector and a capillary column CP-Sil8-CB (5%-diphenyl–95%-dimethyl polysiloxan, Agilent technologies, CA, CA, USA) of 30 m × 0.25 mm i.d., 0.25 µm film thickness, using the same conditions of the gas chromatography-mass spectrometer (GC-MS). The relative number of individual components of oil were calculated by the GC peak and arranged in order of GC elution. EOs constituents were identified based on the retention indices relative to C_5_-C_28_ *n*-alkanes obtained in the same conditions and by comparing their mass spectra with those recorded in the Wiley 7 n.L and those reported in the literature [18].

### 2.4. Phenolic Compounds

#### 2.4.1. Preparation of Plant Extracts

One gram of powdered dried leaves and 5 mL methanol (70%) were transferred to a centrifuge tube and shaken at 120 rpm for 24 h. Then, the samples were centrifuged at 4000 rpm for 15 min, and the supernatant was used for quantification of phenolic compounds. The obtained methanolic extract was diluted with deionized water at a ratio of 1 to 30 and stored at −20 °C. 

#### 2.4.2. Total Phenolic Content

Total phenolic content (TPC) was determined by the spectrophotometric method using the Folin-Ciocalteu reagent described by Wojdylo et al. [19]. Briefly, the methanolic extract (100 µL) was mixed with 200 µL of Folin-Ciocalteu’s reagent (50%) and 2 mL of deionized water in a test tube. After 3 min, 1 mL of 20% Na_2_CO_3_ solution was added to the test tube and vortexed well. The mixture was maintained for 1 h at room temperature in the dark. The absorbance of the samples was recorded at 765 nm using a spectrophotometer (Shimadzu UV-1201, Kyoto, Japan). The concentration of total phenolics was calculated based on a standard curve of gallic acid (0, 50, 100, 150, 200, 250, 300, and 350 μg/mL) as a reference and expressed as mg GAE (gallic acid equivalents)/g of dry weight.

#### 2.4.3. Total Flavonoid Content

The determination of total flavonoid content (TFC) was performed by the aluminum chloride colorimetric method [20]. For this purpose, 1 mL of the extracted sample solution was blended with 300 µL of NaNO_2_ solution (5%). After 5 min, 600 µL of AlCl_3_ (10%) was added to the reaction mixture that was allowed to remain for 6 min. Then, NaOH (4 mL, 1 M) was added to the sample solution and adjusted to 10 mL with distilled water. The absorbance was measured at 510 nm using a spectrophotometer. A calibration curve was constructed with quercetin solutions at concentrations 0 to 1000 μg/mL, and TFC was expressed in term of mg QUE (quercetin equivalents)/g of dry weight.

#### 2.4.4. Total Flavone and Flavanol Contents

The total flavones and flavanols were assayed according to the modified method of Popova et al. [21]. In brief, the methanolic extract (1 mL) was mixed with 1 mL of AlCl_3_ (5%), and the sample solution was adjusted to 2.5 mL with methanol (70%). The absorbance of samples was recorded at 425 nm using a spectrophotometer (Shimadzu UV-1201). Quercetin solution (0–35 μg/mL) was used as a reference standard, and total flavone and flavanol content was expressed as mg QUE/g of dry weight.

### 2.5. Total Anthocyanin Content

The assessment of total anthocyanin content (TAC) content was performed by the pH differential method [22]. Fresh leaves (3 g) were extracted with 20 mL solvent methanol: 10 N HCL (90:10%, *v/v*). The obtained extract was shaken at 200 rpm for 10 min, and the sample solution was centrifuged at 5000 rpm for 15 min at 4 °C. Then, 4 mL supernatant were separated and diluted with 36 mL of two different buffers; potassium chloride pH = 1.0 (0.025 M) and sodium acetate pH = 4.5 (0.4 M), respectively. After 20 min of incubation at room temperature and dark, the absorbance of samples was measured at 520 and 700 nm using a spectrophotometer (Shimadzu UV-1201). The concentration of anthocyanin calculated using Equations (1) and (2).

A = (A_520_ − A_700_) pH_1.0_ − (A_520_ − A_700_) pH_4.5_ (1)

TAC = (A × MW × DF × 100)/MA(2)

where, A refers to the absorbance; MW is molecular weight of cyanidin-3-glucoside (C3G) (449.2 g/mol); DF is the dilution factor (10); MA is the molar absorptivity coefficient of cyanidin-3-glucoside (26,900 M^−1^cm^−1^), and TAC expressed as mg C3G/100 mL of plant extract.

### 2.6. Antioxidant Activity

The antioxidant activity of the extracts was estimated by the reduction of free radical DPPH (2,2-diphenyl-1-picrylhydrazyl) method, as described by Oke et al. [23] with minor modifications. The methanol extract (100 µL) was mixed with 5 mL of 0.004% methanol solution of DPPH radical. The reaction mixture was vigorously shaken, then incubated for 30 min in the dark at room temperature. The absorbance was read against a blank (methanol) at 517 nm using a Shimadzu UV-1201 spectrophotometer, and the percentage of antioxidant activity was calculated as Equation (3).

AA = [(A_B_ − A_S_)/A_B_] × 100 (3)

where, AA is antioxidant activity expressed as a percentage, A_B_ is absorbance of the blank (containing reaction mixture without sample extract) and A_S_ is absorbance of the reaction mixture with sample extract.

### 2.7. Statistical Analysis

All the experiments were performed in triplicate and data presented in this paper were statistically analyzed using completely randomized design (CRD) using SAS version 9.1 (SAS Institute Inc., Cary, NC, USA). When ANOVA *F* test was significant, Duncan’s multiple range test was performed to determine the differences among the mean values at 5% level of significance and results are expressed as the mean ± standard deviation (SD).

## 3. Results

### 3.1. Essential Oil Content

**Figure 1:**
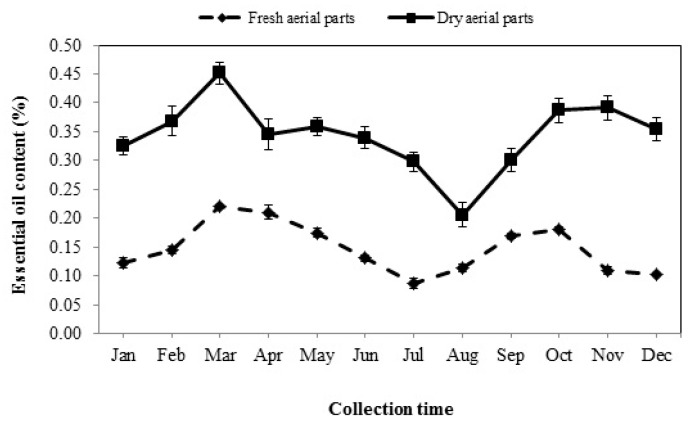
The seasonal variation of the essential oil content extracted from fresh and dried aerial parts of Arabian lilac. Values are the mean ± standard deviation (SD) of three replications (*n* = 3).

The results demonstrated that the EO content underwent profound changes that seemed to strongly depend on the collection time (*p* < 0.01). EOs of the fresh aerial parts showed two intermittent ascendant trends during the year. The plants collected in spring and autumn contained a higher amount of EOs than the other seasons (Figure 1). The oil content of these samples began to increase from January and reached the highest amount (0.22%) at the beginning of the heat period (March). After that, the accumulation of EO gradually decreased from April, and the lowest amount was obtained in July (0.09%). There was a significant difference between the oil content of the plants collected from August to December. Similarly, the accumulation of oils increased again with a slight slope in August and September and showed the second peak in October (0.18%). Then, the percentage of EO declined from November and eventually reached its lowest level in December (0.10%). The dried aerial parts of plants contained more EOs than fresh ones varying in the range of 0.21 to 0.45%. Moreover, the essential oil content of the dried aerial parts significantly changed during the different seasons (*p* < 0.01) and showed a pattern similar to the one reported for the fresh aerial parts (Figure 1). The maximum amount of oils was detected in March (0.45%), followed by October and November (0.39%) while the minimum was recorded in August (0.21%).

### 3.2. Essential Oil Composition

The EO composition of the fresh and dried aerial parts of Arabian lilac, along with their relative percentages and chemical classes in different collection periods are listed in Table 2. According to the GC-MS analysis, forty-one components were identified during different months, representing 97.02 to 98.92% and 97.03 to 99.96% of the fresh and dry oils, respectively. The major components of oils were classified into five groups; monoterpene hydrocarbons (α-pinene and sabinene), sesquiterpene hydrocarbons (β-caryophyllene and laurenene), oxygenated sesquiterpenes (caryophyllene oxide and (5E,9Z)-farnesyl-acetone), diterpene hydrocarbons (phytane and abietadiene) and oxygenated diterpenes (phytol, manool oxide, (6Z,10Z)-Pseudo phytol, manool, and 7α-hydroxy-manool). 

The predominant compounds of the fresh and dried plant’s oils were β-caryophyllene (22.60–35.03% and 25.14–32.43%, respectively), sabinene (7.24–16.73% and 11.04–18.38%, respectively), caryophyllene oxide (3.78–6.38% and 4.48–7.29%, respectively), (6Z,10Z)-Pseudo phytol (0.00–15.02% and 0.00–14.12%, respectively), laurenene (3.29–7.32% and 2.80–6.90%, respectively), (5E,9Z)-farnesyl-acetone (3.03–4.76% and 3.22–5.01%, respectively).

Sabinene, caryophyllene oxide, (6Z,10Z)-Pseudo phytol, (5E,9Z)-farnesyl-aceton, phytol, and α-pinene in fresh oils were less than in dried ones. In contrast, the fresh oils contained higher amounts of the β-caryophyllene, laurenene, phytane, manool oxide, and abietadiene compared with dried aerial parts. The most abundant chemical groups of the total fresh and dried sample’s oils were sesquiterpenes, diterpenes, and monoterpenes. However, the fresh oils were richer in sesquiterpene and diterpene hydrocarbons, and the dry oils contained higher amounts of the oxygenated types of diterpenes and sesquiterpenes.

The data presented in Table 2 clearly shows a high variability in the percentages of the EO composition throughout the year. The biosynthesis of β-caryophyllene, as a major constituent of Arabian lilac’s oil, markedly varied in different months and its annual fluctuation trend was the same for both fresh and dried oils. β-Caryophyllene in fresh oils had an ascendant trend from January, and the highest level was recorded in March (35.03%). Then, there was a clear decline during April and May. Moreover, the lowest level was obtained in June (22.60%). The amount of β-caryophyllene of dry oils increased continuously from February and declined again after rising in September. Furthermore, the maximum and minimum percentage of β-caryophyllene in dry oils were recorded in September (32.43%) and February (25.14%), respectively.

Sabinene, the second main component of Arabian lilac’s oil, was highly increased in spring and autumn in fresh and dried material’s oils. In contrary, there was a great decrease in its amount when oils were harvested during winter and summer. The highest content of sabinene in fresh and dried samples was found in March (16.73%) and October (18.38%), respectively. Additionally, the lowest amounts were obtained in July (7.24%) and August oils (11.04%), respectively (Table 2). The variation pattern of laurenene, manool oxide, manool, α-pinene, and abietadiene of fresh and dried aerial parts’ oils was similar to that exhibited for β-caryophyllene and sabinene.

A quite different trend in the amount of caryophyllene oxide in fresh and dried sample’s oils from other components was found. The amount of this constituent in the fresh plant’s oils of summer and winter seasons was higher than in spring and autumn seasons and the maximum content was obtained in January (6.38%). The maximum of caryophyllene oxide of dried sample oils was observed in June (7.29%) whereas the least amount was obtained when plants were collected in September (4.48%). Similarly, the variations in the amount of (5E,9Z)-farnesyl-aceton, (6Z,10Z)-Pseudo phytol, phytol, and 7α-hydroxy-manool throughout the year was the same as those of caryophyllene oxide changes.

### 3.3. Total Phenolic Content

**Figure 2:**
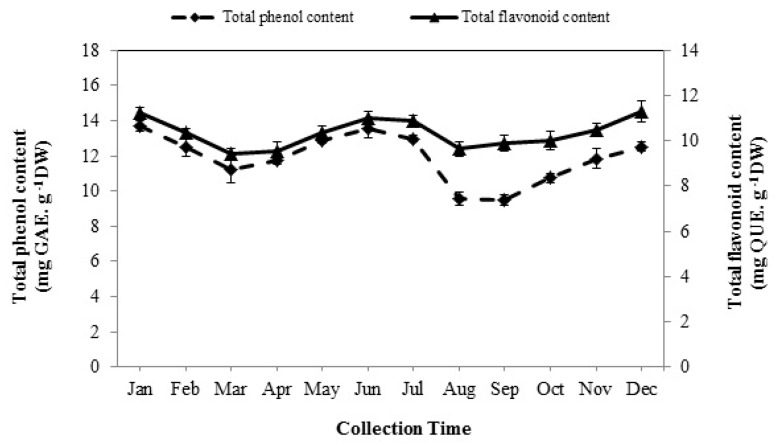
The seasonal variation of total phenol and flavonoid contents of Arabian lilac. Values are the mean ± SD of three replications (*n* = 3). DW: dry weight; GAE: gallic acid equivalents.

The total phenolic content (TPC) (*p* < 0.01) significantly varied in different months of the year ranging from 9.48 to 13.69 mg GAE/g of dry weight (DW) (Figure 2). The TPC gradually increased in December and reached its maximum amount in January (13.69 mg GAE/g DW). Then, a slight drop in TPC occurred in February (12.49 mg GAE/g DW) and March (11.19 mg GAE/g DW). Although, there was no significant difference among TPC of March, April, and May extracts, it increased slightly in spring, and the highest content was obtained in early summer (13.52 mg GAE/g DW). TPC dramatically declined from to September (9.48 mg GAE/g DW). The samples collected in autumn contained a lower value of TPC than those collected in other seasons. However, the least amount was recorded in September (9.48 mg GAE/g DW).

### 3.4. Total Flavonoid Content

The quantitative analysis of total flavonoid content (TFC) showed a significant difference in the diverse seasons (*p* < 0.01). As shown in Figure 2, there was a high level of TFC when the plants were harvested in the winter and summer, while its amount was low during the spring and autumn seasons. The maximum amount of TFC was found in December (11.31 mg QUE/g DW) and remained constant in January and, then, clearly decreased from the end of the winter season (February) and finally reached the minimum level in the middle of the spring (March) (9.40 mg QUE/g DW). Afterwards, a considerable rise occurred in June (11 mg QUE/g DW) which followed on July (10.92 mg QUE/g DW). However, this rate remained unchanged, and it began to increase again from September to December after a sharp drop in August (9.64 mg QUE/g DW).

### 3.5. Total Flavone and Flavanol Contents

**Figure 3:**
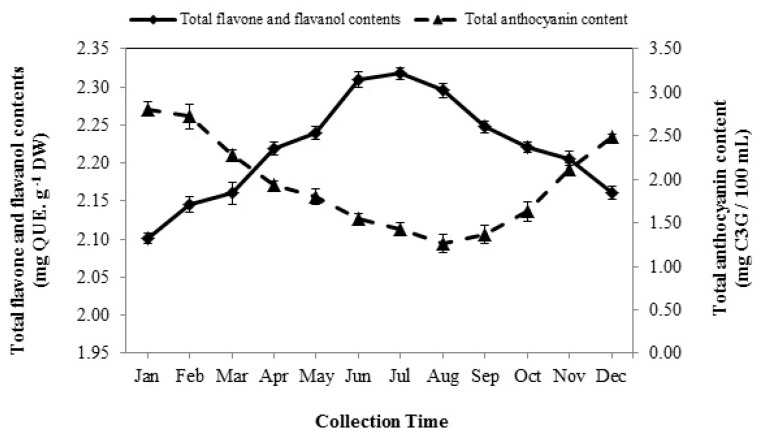
The seasonal variation of total flavone and flavanol, and anthocyanin content of Arabian lilac. Values are the mean ± SD of three replications (*n* = 3). QUE: quercetin equivalents.

The seasonal variation had marked impact on the amount of total flavone and flavanol contents (TFFC) (*p* < 0.01). The lowest concentration of TFFC was recorded in January (2.10 mg QUE/g DW). The amount of the TFFC was continuously increased from February, and the maximum concentration appeared in July (2.32 mg QUE/g DW). Thereafter, an opposite trend was observed and markedly declined from August to December (2.29–2.16 mg QUE/g DW) (Figure 3).

### 3.6. Total Anthocyanin Content

Total anthocyanin content (TAC) significantly differed (*p* < 0.01) from one month to another (1.26–2.81 mg C3G/100 mL). The pattern of seasonal variation of the TAC was interestingly quite reverse with respect to the TFFC pattern. The plants collected in winter contained higher levels of TAC compared with other seasons, and the maximum amount was observed in January (2.81 mg C3G/100 mL). Conversely, the anthocyanins were in lower concentrations during spring and especially summer. As shown in Figure 3, it declined from February to August with relatively sharp slope and the least level was obtained when the plants were harvested in August (1.26 mg C3G/100 mL), which was 2-fold less than that obtained in January. Then, the TAC increased during autumn from September to November.

### 3.7. Total Antioxidant Activity

**Figure 4:**
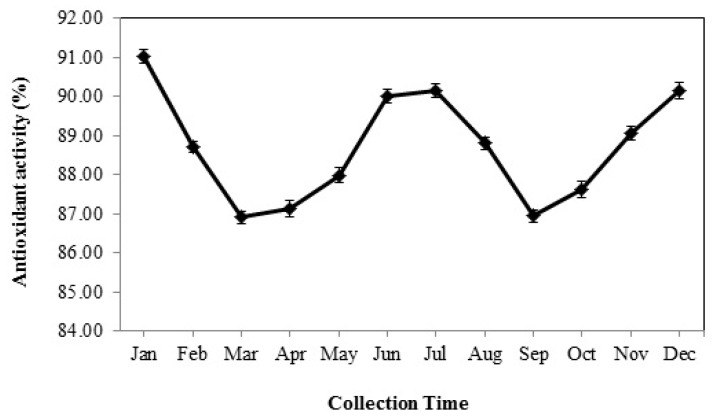
The seasonal variation of antioxidant activity of Arabian lilac. Values are the mean ± SD of three replications (*n* = 3).

Although the extracts wholly exhibited an effective reducing power of the radical species target DPPH^•^, their scavenging effect was strongly dependent on the time of collection (*p* < 0.01). The strongest antiradical potential was detected in January (91.02%) which was not constant, and it significantly decreased from February, and the extracts collected in March showed lower total antioxidant activity (TAA) (86.90%). The TAA of the extracts began to increase during spring and summer and peaked in July (90.15%). After that, a significant decrease trend was observed in the ability to scavenge free radicals of the extracts harvested in August and September. The TAA increased again from October to January (Figure 4). Eventually, the results obtained from the evaluation of DPPH^•^ reducing power indicated that the extracts collected in winter and summer seasons were more effective than the ones harvested in spring and autumn.

## 4. Discussion

In the current study, the EO content extracted from fresh and dried aerial parts of Arabian lilac significantly varied in different collection periods and the samples harvested in spring and autumn showed higher EO contents than those collected during summer and winter. It seems that the increase of EO content (due to defense and protection roles) in the mentioned seasons might be related to the plant's exposure to stress because of a sudden change in environmental and climate conditions in the early spring (when the temperature begins to warm) and early autumn (when temperature begins to cool) (Figure 1). On the other hand, the decrease in EO content during the summer and winter seasons could be due to change in the biosynthetic pathway of secondary metabolites toward the production of metabolites with more defensive potential such as phenolic compounds. Several studies carried out on *Ocimum basilicum* [13], *Mentha spicata* and *Mentha pulegium* [24], and *Artemisia verlotiorum* [25] showed that environmental factors can considerably vary EO content of medicinal plants. 

The variety of the EO’s composition is influenced by some factors including radiation, humidity, soil condition, and temperature. Environmental stresses during seasonal variations can also alter the biosynthetic pathways [26]. In our findings, β-caryophyllene clearly decreased in summer and winter. Since β-caryophyllene is converted to its oxidized form, namely caryophyllene oxide, through an oxidation reaction, the reduction of the level of this component (in fresh and dried aerial parts’ oils) and increase of caryophyllene oxide in these seasons could be ascribed to oxidative stress due to high and low temperature as well as the activation of enzymes of the oxidation reactions. Moreover, the opposite variation patterns of manool with other components of the same class and the reduction of their content in the fresh and dried plant’s oils collected in summer and winter could be attributed to their conversion to oxidized and hydroxylated forms such as manool oxide and 7α-hydroxy-manool, respectively (Table 2). Previously published studies also revealed that EO composition of *Ocimum* species [10,13], *Rosemarinus officinalis* [27], and *Mentha spicata* [24] varies highly throughout the year.

Phenolic compounds, which have several hydroxyl groups, play a notable role in inactivating free radicals and exert antioxidant property [9]. The current study showed the total flavone, and flavanol contents of Arabian lilac significantly varied through the year and extract collected in summer, particularly on July, contained higher amounts of total flavones and flavanols (Figure 3). Regarding the ability of flavonoids to inhibit free radicals and the results obtained in this study, it seems that flavones and flavanols represent a defense mechanism of Arabian lilac against severe heat stress in the summer to reduce the harmful and destructive effects of the oxidative stress caused by heat stress. Our findings confirmed the results of Bujor et al. [16].

According to the literature, this is a first-time evaluation of changes in the TAC during the year. The TAC of the plant obtained in winter was significantly higher than in other seasons (Figure 3). A significant increase in the concentration of anthocyanins in the winter seems to be probably a defense mechanism against low temperature stress to reduce the damage of cell and tissue freezing [28,29,30,31,32,33]. The results interestingly revealed the variations pattern of anthocyanins was opposite with flavones and flavanols. Because the dihydro-flavonol is a common intermediate precursor of anthocyanins, flavones and flavanols [34], it seems that the decrease in temperature during winter has led to a change in the biosynthesis of flavones towards the production of anthocyanins, thereby reducing flavones and increasing anthocyanins.

Moreover, there were two peaks in the quantities of TPC and TFC during the summer and winter while their amount declined significantly in the spring and autumn (Figure 2). The increase in the TPC of extracts collected in summer and winter could be associated to the high quantity of flavones and flavanols content of Arabian lilac in summer and anthocyanins content in winter. Hence, the amount of TPC and TFC decreased in spring and autumn that could be related to a shift in the biosynthetic pathway of secondary metabolites towards the production of EOs in the plant. It seems that when the plant encounters oxidative stress due to a sudden change of temperature in the early spring and autumn, the biosynthetic pathway of secondary metabolites in the plant is directed toward the production of EOs rather than phenolic compounds [34,35].

Furthermore, there are few studies on seasonal variations of the antioxidant properties of medicinal plants. Galasso et al. [11] reported a significant change in the reducing power of *Thymus longicaulis* C. Presl extracts at various harvesting times. The seasonal variations of the antioxidant activity were similar to TPC and TFC (Figure 4). Indeed, the extracts yielded in summer and winter, especially in January and July, with higher levels of flavonoid compounds including flavones and flavanols, showed a higher antiradical power (Figure 2).

## 5. Conclusions

This study provides useful information on the seasonal variations of the quality and quantity of secondary metabolites as well as the antioxidant activity of the Arabian lilac. The results showed that weather conditions in the early spring and autumn were favorable to produce essential oil and increase the amounts of β-caryophyllene, sabinene, and manool. Although EO content decreased during the summer and winter seasons, the plants collected in these seasons contained higher caryophyllene oxide, phytol, (6Z,10Z)-Pseudo phytol, manool oxide and 7α-hydroxy-manool. The optimum time of harvesting plants to achieve the highest quantity of flavones and flavanols was summer, while the samples collected in the winter are rich in anthocyanins. It is recommended to harvest the plant in summer and winter, when it exhibits the highest TPC, TFC, and has the most powerful antioxidant activity. Therefore, the knowledge of the impact of environmental factors, such as seasonal changes, can help producers to choose the best period for plant harvesting and production of plant products richer in the desired compounds for exploitation in pharmaceutical and food industries.

## Figures and Tables

**Table 1:** The air temperature and relative humidity recorded in Iran, Ahvaz locality in 2014.

Month	Temperature (°C)			Relative Humidity (%)		
	Minimum	Maximum	Average	Minimum	Maximum	Average
January	4.6	21	12.8	29	92	61
February	9.6	23.2	16.4	19	64	42
March	15.4	30.4	22.9	27	94	61
April	15.2	28.2	21.7	20	46	33
May	28	37	32.5	19	43	31
June	28.8	45.2	37	8	31	20
July	30	50.2	40.1	12	62	37
August	30.6	48	39.3	20	74	47
September	29.6	44.8	37.2	15	46	31
October	24	35.6	29.8	41	97	69
November	10.6	28.4	19.5	25	73	49
December	14.4	17.6	16	64	95	80

**Table 2:** chemical constituents of Arabian lilac (*Vitex trifolia* var. *purpurea*) essential oils at different collection periods (2014).

				GC area (%) ^a^																							
Compounds	RT ^b^	KI ^c^	KI ^d^	January		February		March		April		May		June		July		August		September		October		November		December	
				FAP	DAP	FAP	DAP	FAP	DAP	FAP	DAP	FAP	DAP	FAP	DAP	FAP	DAP	FAP	DAP	FAP	DAP	FAP	DAP	FAP	DAP	FAP	DAP
Thujene	7.58	933	924	0.15	0.37	0.18	0.38	0.23	0.36	0.29	0.36	0.48	0.60	0.23	0.51	0.17	0.34	0.16	0.27	0.21	0.20	0.26	0.42	0.14	0.35	0.11	0.20
**α-Pinene**	**7.79**	**939**	**932**	**3.27**	**1.35**	**4.10**	**4.00**	**4.29**	**4.62**	**4.40**	**5.20**	**2.45**	**3.22**	**1.77**	**2.29**	**2.00**	**2.56**	**2.15**	**2.55**	**3.18**	**5.35**	**3.61**	**4.86**	**2.79**	**4.06**	**2.95**	**3.66**
**Sabinene**	**9.05**	**975**	**969**	**11.71**	**13.03**	**13.23**	**15.1**	**16.73**	**18.14**	**16.38**	**18.17**	**9.58**	**16.45**	**9.11**	**12.49**	**7.24**	**12.08**	**9.22**	**11.04**	**15.13**	**15.09**	**16.02**	**18.38**	**11.63**	**17.94**	**9.68**	**15.47**
β-Pinene	9.09	976	974	1.54	0.78	2.00	0.80	1.86	1.07	0.47	1.14	0.50	0.96	0.37	0.07	0.31	0.04	0.32	0.03	0.57	0.01	0.87	0.20	1.83	0.86	1.26	0.30
1-Octen-3-ol	9.14	978	974	0.64	2.61	0.71	2.14	-	-	-	-	2.95	-	2.39	1.53	1.89	1.84	0.61	0.87	0.64	-	-	-	2.98	0.32	1.99	2.66
Myrcene	9.43	986	988	0.14	0.85	0.21	0.24	0.28	0.27	0.30	0.85	0.11	0.20	0.10	0.10	-	-	-	-	0.14	-	0.30	0.42	0.13	0.32	-	0.16
α-Terpinene	10.16	1009	1014	0.83	0.44	0.27	0.17	-	0.14	-	0.15	0.39	0.42	0.41	0.49	0.48	0.35	0.15	0.53	-	0.19	-	0.27	0.21	0.45	0.20	-
p-Cymene	10.39	1017	1020	-	0.47	0.24	0.75	0.42	0.78	0.55	0.53	0.28	0.78	0.24	0.37	0.16	0.27	-	-	0.21	-	-	1.07	0.15	0.51	0.10	-
β-Phellandrene	10.52	1021	1025	-	0.26	0.25	0.12	0.33	0.18	0.14	0.23	0.27	0.29	0.39	0.26	0.17	0.18	0.17	0.28	0.21	0.19	0.39	0.26	0.33	0.43	0.16	0.30
1,8-Cineole	10.60	1024	1026	0.10	0.19	0.22	-	0.16	-	-	0.12	0.25	0.21	0.21	0.23	0.24	0.18	0.14	0.31	0.16	0.14	0.27	0.23	0.26	0.19	-	0.22
γ-Terpinene	11.42	1052	1054	-	2.12	1.39	0.88	-	0.97	0.99	0.53	2.03	1.44	1.93	0.68	1.49	0.33	0.57	0.58	0.64	0.06	1.37	2.17	1.66	2.68	1.06	2.68
α-Terpinolene	12.28	1081	1086	-	0.22	-	0.26	-	0.12	-	0.09	0.10	0.10	0.08	-	-	-	-	-	-	-	0.11	0.18	0.35	0.24	-	-
Terpinen-4-ol	14.93	1171	1174	0.75	-	0.47	-	0.16	-	0.16	-	0.16	-	0.68	-	0.70	-	0.57	-	0.47	0.03	0.50	-	0.62	-	0.16	-
Estragol	15.48	1190	1195	-	2.72	-	1.68	0.63	-	-	-	2.88	-	1.33	-	1.76	-	-	0.98	1.68	-	-	-	0.98	-	0.27	2.87
Bornyl acetate	17.93	1280	1284	0.16	-	0.15	0.14	0.20	-	-	-	-	-	0.15	0.12	0.16	0.11	-	0.14	-	0.09	-	0.13	0.10	-	0.14	-
α-Terpinyl acetate	19.63	1343	1346	0.30	0.51	0.42	0.33	0.18	-	0.33	0.41	0.38	0.36	0.28	0.33	0.31	0.54	0.29	0.20	0.27	0.18	0.25	0.23	0.31	0.53	0.33	0.46
**β-Caryophyllene**	**21.71**	**1422**	**1418**	**25.76**	**25.29**	**26.33**	**25.14**	**35.03**	**27.51**	**34.41**	**29.39**	**24.32**	**28.80**	**22.6**	**26.03**	**24.01**	**26.71**	**32.49**	**27.46**	**34.16**	**32.43**	**31.81**	**31.99**	**26.44**	**28.76**	**27.36**	**27.03**
α-Caryophyllene	22.37	1450	1454	0.91	0.85	1.26	0.97	1.21	1.20	1.04	1.16	1.07	1.15	0.91	1.23	0.87	1.18	1.35	0.97	1.44	1.19	1.20	1.18	1.00	1.13	1.01	1.19
Germacrene-D	23.01	1476	1484	-	-	-	-	-	-	0.16	-	0.93	-	0.14	0.19	0.20	0.25	0.26	0.34	0.16	-	0.12	-	-	-	-	-
β-Selinene	23.14	1481	1489	-	0.10	-	-	-	-	-	-	0.78	-	0.53	0.21	0.23	0.29	0.42	0.38	0.25	-	-	-	-	-	0.17	-
α-Selinene	23.35	1490	1498	0.64	0.59	0.79	0.59	0.60	0.86	0.79	0.85	1.53	0.85	0.33	0.36	0.79	0.44	0.37	0.58	0.91	0.42	0.43	0.59	0.53	0.66	0.66	0.85
E-Nerolidol	24.91	1554	1561	1.18	-	-	0.11	-	0.16	-	-	0.33	0.15	0.22	0.17	0.19	0.19	0.23	0.13	0.22	0.10	-	-	0.14	-	0.11	-
**Caryophyllene oxide**	**25.47**	**1578**	**1582**	**6.38**	**6.89**	**4.74**	**6.83**	**3.78**	**6.35**	**3.93**	**6.33**	**4.23**	**7.05**	**5.46**	**7.29**	**6.24**	**6.75**	**5.98**	**4.67**	**3.91**	**4.48**	**3.87**	**4.59**	**4.54**	**4.96**	**4.77**	**5.76**
Caryophylladienol II	26.57	1626	1631	0.61	0.66	-	-	0.22	0.13	0.38	-	0.14	0.13	0.40	0.43	0.31	0.36	0.30	0.70	-	0.33	0.45	0.55	0.33	0.40	0.37	0.74
(Z)-14-hydroxy-Caryophyllene	27.41	1664	1666	0.91	1.82	0.71	0.63	0.42	-	0.58	-	1.81	0.75	2.46	1.34	2.75	1.25	2.63	0.87	1.85	0.62	0.45	0.52	0.39	0.72	0.55	0.71
**Phytane**	**30.12**	**1789**	**1792**	**2.73**	**3.03**	**2.69**	**2.56**	**3.98**	**2.80**	**3.75**	**2.38**	**4.45**	**2.54**	**3.96**	**3.84**	**4.30**	**3.68**	**4.42**	**3.41**	**4.71**	**3.01**	**3.68**	**2.32**	**3.19**	**2.83**	**3.71**	**3.51**
**(5E,9Z)-Farnesyl acetone**	**31.93**	**1879**	**1886**	**4.76**	**5.01**	**4.49**	**4.10**	**3.12**	**3.22**	**3.41**	**3.29**	**3.23**	**3.37**	**4.25**	**4.11**	**4.61**	**4.42**	**4.49**	**4.88**	**3.55**	**4.28**	**3.03**	**4.11**	**4.09**	**3.49**	**4.49**	**4.04**
**Laurenene**	**32.26**	**1896**	**1887**	**4.07**	**2.80**	**3.71**	**3.92**	**5.26**	**4.42**	**5.46**	**4.56**	**3.29**	**4.10**	**3.56**	**3.57**	**4.06**	**3.67**	**6.88**	**5.89**	**7.32**	**6.90**	**6.23**	**6.89**	**3.68**	**5.77**	**3.90**	**4.00**
epi-Laurenene	32.46	1906	1901	-	0.71	0.95	0.62	1.88	0.85	1.33	-	-	1.46	1.23	-	-	-	-	1.34	2.94	-	1.53	1.20	1.33	-	1.15	1.08
**Phytol**	**33.26**	**1946**	**1942**	**4.01**	**3.43**	**3.72**	**4.58**	**3.01**	**3.81**	**2.28**	**3.31**	**2.83**	**3.55**	**2.99**	**3.77**	**3.81**	**4.51**	**3.71**	**4.03**	**2.08**	**3.34**	**2.51**	**3.02**	**2.55**	**3.97**	**3.90**	**3.17**
**Manool oxide**	**33.64**	**1965**	**1987**	**1.99**	**0.10**	**6.93**	**0.11**	**8.34**	**7.50**	**7.11**	**6.03**	**1.05**	**5.35**	**0.10**	-	-	**0.10**	**0.10**	**0.11**	**0.31**	**0.10**	**8.01**	**5.05**	**7.21**	**6.42**	**0.10**	**0.10**
**(6Z,10Z)-Pseudo phytol**	**34.03**	**1985**	**1988**	**10.01**	**0.23**	**0.20**	**0.64**	-	-	**0.10**	**6.72**	**0.11**	**8.34**	**10.92**	**12.67**	**15.02**	**14.12**	**9.93**	**13.54**	**0.10**	**13.63**	**0.27**	**0.34**	**0.40**	**0.28**	**10.5**	**0.15**
Manoyl oxide	34.11	1989	1989	2.98	1.99	1.96	0.98	-	-	-	-	2.97	-	1.62	0.38	1.43	0.21	1.32	0.19	1.91	-	1.54	-	1.69	0.58	2.26	1.23
(E,E)-Geranyl linalool	34.88	2031	2026	0.11	1.96	1.97	1.91	-	1.02	-	-	2.99	-	1.92	0.17	1.49	0.19	0.23	0.13	0.22	-	-	-	0.14	-	1.21	1.09
**Manool**	**35.37**	**2058**	**2056**	**1.35**	**0.74**	**1.45**	**1.24**	**1.74**	**1.45**	**1.67**	**1.30**	**1.50**	**1.32**	**1.37**	**0.60**	-	**0.90**	**0.10**	**0.89**	**1.36**	**1.09**	**1.30**	**1.26**	**1.44**	**1.19**	**1.62**	**1.12**
Sclareolide	35.48	2064	2065	-	2.69	3.21	2.03	-	1.97	3.87	-	5.08	0.15	1.27	0.96	1.33	1.13	-	1.07	-	2.02	1.88	1.60	3.44	2.94	-	2.83
1-Octadecanol	35.64	2073	2077	0.56	2.86	0.84	1.98	-	-	-	-	2.99	-	2.95	1.43	2.73	1.92	2.29	1.89	0.55	-	-	0.88	1.99	0.49	0.93	2.48
**Abietadiene**	**35.82**	**2082**	**2087**	**4.12**	-	**1.63**	**3.56**	**2.97**	**4.59**	**2.80**	**4.36**	**2.93**	**4.29**	-	**3.23**	**1.88**	**0.10**	**2.01**	**1.11**	**3.61**	**2.81**	**5.04**	**2.03**	**4.88**	**3.17**	**4.96**	**4.36**
Laurenan-2-one	36.40	2114	2115	0.79	1.97	1.63	0.94	-	-	-	-	2.04	-	3.08	0.20	0.75	0.26	0.58	0.29	0.52	-	0.37	-	0.47	-	0.57	0.26
**7α-hydroxy-Manool**	**38.62**	**2240**	**2237**	**1.58**	**3.06**	**0.53**	**1.73**	-	**0.31**	**0.11**	**1.58**	**2.25**	**1.58**	**2.92**	**4.18**	**1.63**	**2.80**	**0.93**	**2.48**	**0.36**	**1.66**	**0.31**	**1.97**	**0.35**	**1.28**	**1.42**	**2.07**
Dehydroabietal	39.22	2276	2263	2.04	4.52	3.76	4.87	-	3.17	-	0.40	2.94	-	2.16	1.90	1.43	2.83	1.72	1.91	1.07	-	0.94	-	2.90	0.51	2.92	0.38
Chemical class																											
Monoterpene hydrocarbons				17.64	19.89	21.87	22.70	24.14	26.65	23.52	27.25	16.19	24.46	14.63	17.26	12.02	16.15	12.74	15.28	20.29	21.09	22.93	28.23	19.22	27.84	15.52	22.77
Oxygenated monoterpenes				1.31	0.70	1.26	0.47	0.70	-	0.49	0.53	0.79	0.57	1.32	0.68	1.41	0.83	1.00	0.65	0.90	0.44	1.02	0.59	1.29	0.72	0.63	0.68
Total monoterpenes				18.95	20.59	23.13	23.17	24.84	26.65	24.01	27.78	16.98	25.03	15.95	17.94	13.43	16.98	13.74	15.93	21.19	21.53	23.95	28.82	20.51	28.56	16.15	23.45
Sesquiterpene hydrocarbons				31.38	30.34	33.04	31.24	43.98	34.84	43.19	35.96	31.92	36.36	29.30	31.59	30.16	32.54	41.77	36.96	47.18	40.94	41.32	41.85	32.98	36.32	34.25	34.15
Oxygenated sesquiterpenes				14.63	19.04	14.78	14.64	7.54	11.83	12.17	9.62	16.86	11.60	17.14	14.50	16.18	14.36	14.21	12.61	10.05	11.83	10.05	11.37	13.40	12.51	10.86	14.34
Total sesquiterpenes				46.01	49.38	47.82	45.88	51.52	46.67	55.36	45.58	48.78	47.96	46.44	46.09	46.34	46.90	55.98	49.57	57.23	52.77	51.37	53.22	46.38	48.83	45.11	48.49
Diterpene hydrocarbons				6.85	3.03	4.32	6.12	6.95	7.39	6.55	6.74	7.38	6.83	3.96	7.07	6.18	3.78	6.43	4.52	8.32	5.82	8.72	4.35	8.07	6.00	8.67	7.87
Oxygenated diterpenes				24.07	16.03	20.52	16.06	13.09	17.26	11.27	19.34	16.64	20.14	24.00	23.67	24.81	25.66	18.04	23.27	7.41	19.82	14.88	11.64	16.68	14.23	23.93	9.31
Total diterpenes				30.92	19.06	24.84	22.18	20.04	24.65	17.82	26.08	24.02	26.97	27.96	30.74	30.99	29.44	24.47	27.79	15.73	25.64	23.6	15.99	24.75	20.23	32.60	17.18
Phenylpropanoids				-	2.72	-	1.68	0.63	-	-	-	2.88	-	1.33	-	1.76	-	-	0.98	1.68	-	-	-	0.98	-	0.27	2.87
Alcohols				1.20	5.47	1.55	4.12	-	-	-	-	5.94	-	5.34	2.96	4.62	3.76	2.90	2.76	1.19	-	-	0.88	4.97	0.81	2.92	5.14
Total Identified				97.08	97.22	97.34	97.03	97.03	97.97	97.19	99.44	98.6	99.96	97.02	97.73	97.14	97.08	97.09	97.03	97.02	99.94	98.92	98.91	97.59	98.43	97.05	97.13
Not-identified				2.92	2.78	2.66	2.97	2.97	2.03	2.81	0.56	1.40	0.04	2.98	2.27	2.86	2.92	2.91	2.97	2.98	0.06	1.08	1.09	2.41	1.57	2.95	2.87

^a^ Percentages obtained by FID peak-area normalization; RT ^b^ = Retention time; KI ^c^ = Kovats index; KI ^d^ = Kovats index in the literatures; FAP = Fresh aerial parts of plant; “-“ = Not detected; DAP = Dried aerial parts of plant. The bold values are the main components of essential oil.
